# The Water Status in China and an Adaptive Governance Frame for Water Management

**DOI:** 10.3390/ijerph17062085

**Published:** 2020-03-21

**Authors:** Jiahong Li, Xiaohui Lei, Yu Qiao, Aiqing Kang, Peiru Yan

**Affiliations:** 1 School of Civil Engineering, Tianjin University, Tianjin 300354, China; 2 Institute of Water Resources and Hydropower Research, Beijing 100038, China; 3 Construction and Administration Bureau of South-to-North Water Diversion Middle Route Project, Beijing 100038, China

**Keywords:** water status, adaptive governance, water management frame, adaptive water resource management

## Abstract

China is increasingly facing water-related problems, such as water scarcity, pollution, and overexploitation of groundwater. This paper discusses the water status in China and claims that governance is the cause of water-related problems. The structure of the current water management is analyzed to conclude that the control-command is a static approach which is less capable of dealing with the uncertainty in the water resources system. An adaptive governance frame is introduced, which highlights the learning process and participation. The learning process avoids making the same mistake twice and the participation ensures the diversity of information, which are both necessary for water resources management.

## 1. Introduction

Sustainable water management is of fundamental importance for society but remains an extraordinary challenge across the world, with many pressing issues to be addressed, such as the lack of sanitation, the depletion of water resources, and the financial loss associated with hydrological extremes such as floods and droughts [1]. Moreover, this ongoing problem of water resources management has been expedited by the prospect of climate and global change [2] as it can change the availability and distribution of freshwater, as well as the demand for water [3] and groundwater availability [4].

Traditional resource governance frames have evolved over a long period and are closely intertwined with technological infrastructure [5,6,7]. However, in the last quarter of the 20th century, public discourse has questioned the desirability of relying solely on the scientific rationalist approach to manage and use the natural resources [8]. The traditional water resource management is generally referred to as supply management. Its main feature is to achieve the balance between water resource supply and demand by developing new water sources and large-scale long-distance water transfer. The supply management depends on the perspective of the “endless water,” which ignores the possibility of saving water in management. The traditional decision-making method is top-down and cannot adequately reflect the changes in the demands of water users [9]. The increase of water waste and water inefficiency has resulted from the extensive management method. With the intensification of the contradiction between supply and demand of water resources, the ability to rely solely on the increase of water conservancy projects to solve water shortage problems is increasingly restricted [10]. Therefore, the transformation of water resource management methods and demand management methods has emerged. Water demand management is centered on water users, and the decision-making method is bottom-up. It can adequately reflect the water use level and efficiency of water users and improve and coordinate the behavior of water users, achieving the goals of water management.

Resource managers underestimated the consequences of feedback effects, non-linearity, time delays, and changes in human behavior, which are important for policy making. Increasing recognition of economic and social uncertainties and policy complexities has prompted a widespread reconsideration of management and governance. It has also become evident that many problems are not primarily associated with the resource base but must be attributed to governance failures [2]. Adger et al. [11] and Rijke [12] claimed that developing resilient water resource management systems is more a governance issue than a technological issue as “adaptation to climate change is limited by the values, perceptions, processes and power structures within society’’. Emmanuel et al. [13] established a theoretical framework for sustainable water resource management and framed a strategy which reconciles demand and supply of water while considering environmental, economic, and social interests. Critical voices have argued for major paradigm shifts in management [14,15]. The ability of governance systems to deal with uncertainty and surprise is an essential requirement for sustainability when facing off the increasing uncertainty due to climate and global change [2]. Several types of research have also been led to suggest adaptive governance of water resources in China [16,17,18]. Adaptation measures in water resource management policies and practices are necessary to meet the challenges of the current and upcoming climate change. Both supply-side and demand-side adaptation strategies can be taken to ensure water supply during average and drought conditions. Meanwhile, climate change affects the function and operation of existing water-related infrastructure, including hydropower, structural flood defenses, drainage and irrigation systems, as well as water management practices [19]. Climate changes influence runoff in wet seasons, and the effect of climate changes on fish habitat quality in lower reach is high in dry seasons but low in flood seasons [20]. In China, water resource scarcity and widespread water pollution, combined with the increasing demands resulting from the rapid social and economic growth, limit its further development and sustainability [21,22,23,24]. Water supply shortages in China could also threaten global sustainable development and have worldwide impacts if the country’s ability to produce sufficient food to feed a large and growing population can no longer be maintained [25,26]. Consequently, improvements in water governance present a crucial challenge for better water management.

Management should not only guarantee the services provided by the resource (e.g., water for irrigation, fisheries), prevent damage (e.g., flooding), and maintain the use for future generations (e.g., preserve groundwater resource), but also respect the integrity of ecosystems as a goal (e.g., maintenance of a good ecology) [27]. The concept of Adaptive Water Resource Management (AWRM) was developed to manage these multiple stresses and demands on the water environment better. It promotes better coordination between the activities of individual citizens, businesses, and organizations involved in water relevant policies [1]. It helps to handle the contradiction that the current management frame may be successful in the short term, but the adverse effects of long-term consequences may often outweigh the short-term benefits [27].

Water resource management is mainly the responsibility of the government, according to law in China. The development and evolution of China’s water resource management and protection frame have experienced four phases: (1) informal water resources management in engineering, (2) the formal system of executive orders, (3) the management of water abstraction permits, and (4) the management of formal systems based on water rights. Various supply management approaches, such as building large-scale infrastructures of water storage and long-distance, inter-basin transfers, and development of alternative water resources through rainwater harvesting, precipitation enhancement, desalination, and wastewater reclamation, have been carried out to balance the spatial and temporal availability and to increase the supply of water resources [21].

Management refers to activities of analyzing, monitoring, developing, and implementing measures to keep the state of a water resource within desirable bounds [28]. Adaptive management accepts the uncertainties of resource management and attempts to sort these uncertainties over time by a process of using management actions as experiments to test policy [29]. Adaptive management is a scientific or technical approach to resource management, but often requires a specific set of social, economic, and governance factors for implementation [30].

Adaptive governance is an experimental approach used by water management agencies around the world to manage and restore aquatic ecosystems. The effectiveness of the approach can often be constrained by an inflexible management frame [31]. Adem and Geneletti et al. [32] investigated a case study of knowledge co-production, to assess the applicability of boundary work as a conceptual framework to support implementing adaptive governance in the water sector. A novel operation chart for a cascade hydropower system has been presented to alleviate ecological degradation in hydrological extremes [33]. Ha and Dieperink et al. [34] published a paper with recommendations both for national, regional, and local policy interventions and for future adaptive freshwater management. Management reform emphasizing adaption can contribute to water security and yield socio-economic outcomes through a systemic understanding of how the water system functions, and by connecting goals and budgets across multiple, currently fragmented policy areas [35]. Using an informal adaptive governance framework, Wolfenden and Wassens et al. [36] delivered return flows from a forested wetland complex into a large lowland river in south-eastern Australia. Combining with the advanced concepts and the status of water resources management in China, this paper puts forward a suitable adaptive governance frame for water resources to maintain and improve the resilience of water resource systems in China; that is, the continuity and adaptability of water resource systems to external disturbances. Adaptive water management is not only an optimization of resources, but also the optimization of management capabilities.

What distinguishes adaptive governance from adaptive management is the functional and geographical scope of institutional activities. Adaptive governance makes an obvious connection between the multilevel governance of natural resources as an important component of the overall institutional mechanisms for decision making and implementation in wider society [37].

Hence this paper seeks to provide a set of composite policies or patterns that address and integrate different problem domains. Leadership, multiplicity, changeability in competencies and timing seem to be key factors in adaptive governance. In recent years, with the rapid development of society and the economy, China’s water resources have become increasingly scarce, so the competition among various sectors for water resources has become increasingly fierce. However, water pollution has become very serious. How to carry out the efficient and sustainable use of water resources has become the primary task of water resource management. Water resources form a complex giant system with multiple uncertainties within it. The planning and management of water resource systems face multiple contradictions and conflicts. The previous command and control water management paradigm cannot coordinate the economic development and ecological environment problems well in the utilization of water resources. The current so-called "command-oriented" management model cannot respond to changes in the environment and ecology in a timely manner [38,39]. This type of planning model often fails to cope with unexpected conditions and crises [40,41,42]. The traditional management model can be regarded as a method focused on maximizing output based on a standard economic model, and its structure often remains unchanged for a long time, which tends to focus on formal administrative structures, those being explicitly stated in policy documents and laws, despite the acknowledgment that these may have little to do with the everyday reality of natural resource management [43]. In many cases, there are often conflicts and contradictions between different goals, and the maximization of short-term goals often comes at the cost of ecological damage. One approach to managing complexities and uncertainties within any complex system is termed adaptive governance, commonly used in the study of social-ecological systems [40], and adaptive water resource management can comprehensively consider the ecological, social, economic, and environmental aspects related to water resources, and continuously adjust the feedback from the bottom to the top to solve water resource planning issues. Adaptive water resource governance can be considered as a systematic method for continually improving policies and activities by learning from the outcomes of actions and activities. Water resource adaptive governance arises from the complex adaptive system (CAS), which has complex, non-linear, self-organizing characteristics and can change as the environment changes [44]. The biggest feature of its system is that it can learn actively and learn from past experiences to avoid the same mistakes. Under adaptive governance, the concept of water security is not a fixed, pre-set goal, but rather a dynamic process that will be continuously updated and modified to respond to the everchanging world [38]. The adaptive water resource management can comprehensively consider the ecological, social, economic, and environmental aspects related to water resources, and continuously adjust the feedback to solve water resource planning issues and provide sustainable development for relevant plan decision makers. The leaders must embrace uncertainty and seek opportunities for learning through experimentation. It is important to manage the uncertainties in the social and political relationships, with the experimentation supported by broad-based stakeholders. Adaptive governance is a critical component for solving the problem of water resources.

The structure of this paper is as follows: We first introduce water status and water associated policies and practices in China. Based on the current political, socio-economic and water resources conditions in China, the current water management frame is analyzed. To improve the water resources management frame, the adaptive Water Resource Management (AWRM) theory is analyzed. Finally, we recommend the water resources adaptive governance frame in China.

## 2. The Water Status in China

### 2.1. Water Resources Management in China

#### 2.1.1. Water Resource Management System in China

**Figure 1:**
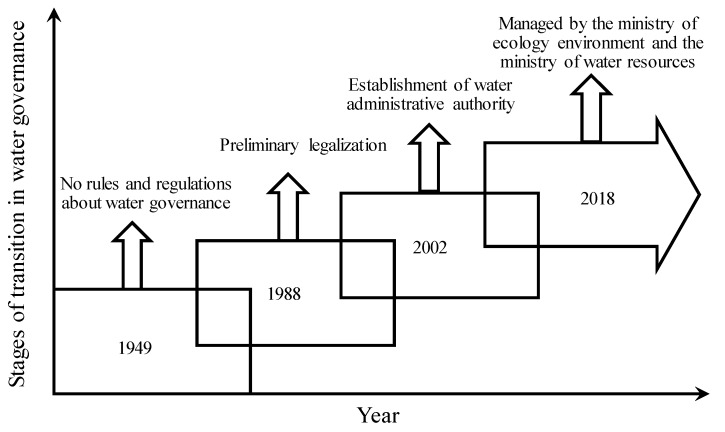
Stages of transition in water governance of China.

Water resource management is mainly the behavior of the government in managing water according to law, managing administrative authority according to law, and the scientific management of water. China’s water resource management system can be summarized in the following stages of development, which can also be seen in the Figure 1.

Before the promulgation of the Water Law in 1988, China implemented a multi-sectoral and decentralized management model, which had different and unclear administrative institutes that worked separately for water resource management. The water resource management system was relatively fragmented. In the absence of a legal definition, the intensity and effectiveness of implementation were relatively poor.

After the promulgation of the Water Law in 1988 and before the promulgation of the new Water Law in 2002, China implemented a management model that combines unified management with grading and sub-sector management, which means the Ministry of Water Resources, provinces, and cities manage the different parts of water resources. China’s water resource management has initially embarked on the track of legalization.

The new Water Law, promulgated in 2002, marked the beginning of a new stage in the construction of China’s water resource management system. The system of combining the major river basin management and regional administrative management of water resources was clarified. The water administrative department of the State Council is responsible for the unified management and supervision of water resources throughout the country.

Although the new "Water Law" has made a significant breakthrough in the reform of water resource management systems, many specific systems are not sound due to the influence of various factors. Moreover, in the current legal environment, the implementation of these imperfect systems is also facing considerable difficulties. Over past years, China has completed legislation for water management reform, especially the revision and promotion of the Water Law in 2002, which covered every aspect of economic and social development. However, the law has not been effectively implemented.

**Figure 2:**
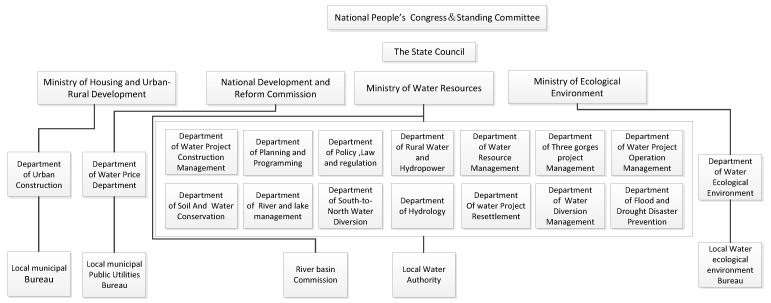
China’s water management framework.

The existing water resource administrative system of China, which was reformed in 2018, is shown in Figure 2. Water resources are managed by both the central and the local governments. However, fragmented issues exist due to the involvement of several agencies. From a national point of view, there are four Ministries in charge of water resource management: The Ministry of Housing and Urban-Rural Development, the National Development and Reform Commission, the Ministry of Water Resources, and the Ministry of Ecological and Environment.

The major policy is “Three Red Lines". The first is to establish a red line for controlling the development and utilization of water resources. By 2030, the total national water consumption will be controlled within 700 billion m^3^. The second is to establish a red line for water use efficiency control. By 2030, water use efficiency will reach or approach the world’s advanced level, water consumption per added value 10,000 yuan of industrial will be reduced to less than 40 m^3^, and the effective utilization coefficient of farmland irrigation water will be increased to above 0.6. The third is to establish a red line for limiting water pollution in water function zones. By 2030, the total amount of major pollutants entering rivers and lakes will be controlled within the water function zone’s ability to accept pollutants, and the water quality zone’s compliance rate will increase to more than 95%.

Such a fragmented administrative system and policies pose a serious challenge to effective and efficient water management. Particularly, due to a lack of a leading agency, none of them are subordinated to one another, meaning the water-related jurisdictions of these involved agencies are not clear. Because sometimes one river does not only belong to one province, and the water resources management problems do not only belong to one agency.

#### 2.1.2. Major River Management Basins in China

Because of the unreasonable water management, the problems of water shortages and degraded water quality are still serious [45]. Water environment protection is another popular topic, and the water-related problem has troubled many places in China for many years. To achieve better management of water resources, the ministry of water resources set up seven affiliated institutions, which are in seven major catchments in China.

**Figure 3:**
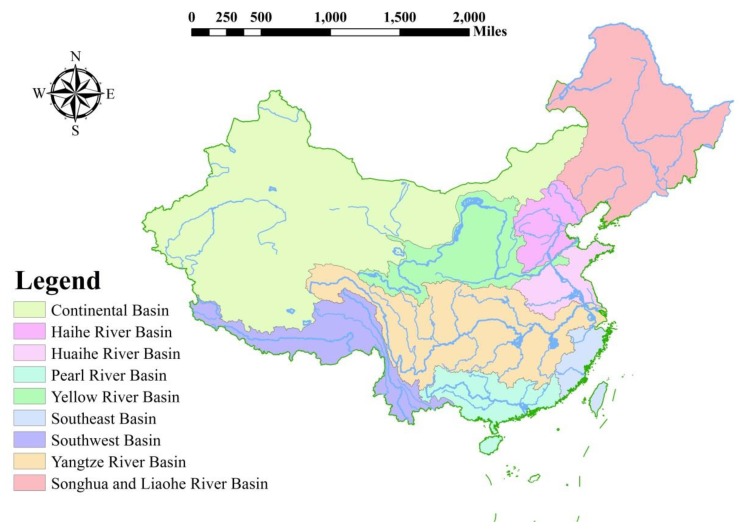
China’s major river basins.

China has more than 1500 rivers, with drainage areas of 1000 km^2^ or greater, and more than 50,000 with a catchment area over 100 km^2^. The majority of these rivers are located in the eastern parts of China where the monsoonal climate produces abundant rainfall. Figure 3 shows the location of China’s major river basins.

After decades of evolution, the current water resource management frame has become a combination of river basin management and administrative region management. From the perspective of the river basin, the ministry of water resource was set up to plan, manage, and allocate all kinds of water resources within each river basin (i.e., surface water and groundwater, etc.). It has the responsibility to develop an integrated water-consuming plan in the middle-term and long-term level and coordinate every province’s interest.

In water resource management, there are some remarkable results that have been achieved. For example, the establishment of an integrated management system that combines watershed management and regional management, the establishment of a water rights management system that focuses on water allocation, water abstraction permits and water resources demonstration, and the establishment of a water price management system based on full cost accounting. There are still problems such as the unclear water resources ownership, and the lack of protection of the water and environmental rights.

### 2.2. Present Status of Water Resources Issues in China

#### 2.2.1. Flood and Drought in China

**Figure 4:**
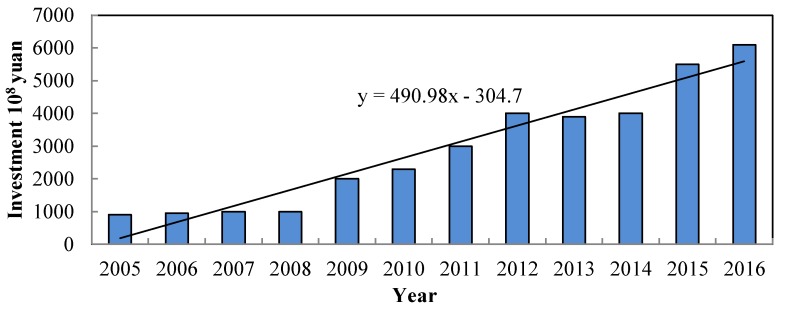
Investment in water infrastructure. Date source: Statistic Bulletin on China Water Activities.

**Figure 5:**
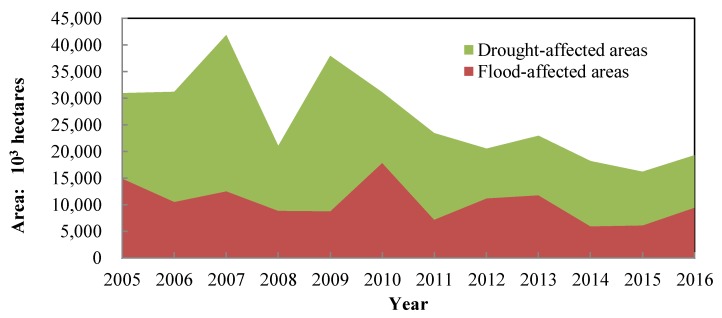
Flood-affected areas and drought-affected areas. Date source: Bulletin of Flood and Drought Disasters in China.

After decades of industrialization, China has achieved tremendous economic growth and development. However, China’s economic growth has come at the cost of resource over-consumption. The total water supply was 6040.2 × 10^8^ m^3^ in 2016, which went up by 7% compared with 5633 × 10^8^ m^3^ in 2005, indicating a significant growth trend. With the request for water safety, the Chinese government has invested much time and money in the construction of water conservancy infrastructure(Figure 4), in the hope of decreasing the frequency of water related disasters. In 2005, only 827.4 × 10^8^ RMB was used for water infrastructure. By the year 2016, investment has grown to 6099.6 × 10^8^ RMB, almost eight times that of 2005. Though so much effort has been put in, it did not turn out as expected, in that the total areas of flood-affected and drought-affected did not reduce obviously (Figure 5). There was not only no significant decline in the disaster area, but also many new water-related issues appearing. Water quality, for example, has become worse and worse. More than two-thirds of lakes have been plagued by different degrees of eutrophication, while more than 850 rivers have been polluted in 1200 monitored rivers [46]. On the other hand, the growing exploitation of groundwater has given rise to grievous consequences. Land subsidence has occurred in 96 cities and regions in China. For example, in the east of Beijing, the average land subsidence was 10 cm each year from 2003–2011 [47], which initiate the intrusion of seawater and other ecological issues.

#### 2.2.2. Low Level of Water Resources and Water Environment

Water scarcity and poor water quality are interwoven with many other things, for instance, economic development, and life quality [45]. In China, agriculture uses the largest proportion of water. As is widely known, China has the largest population in the world (almost 1/5 of the world’s population) and makes up only seven percent of the world’s land. To feed such a large population, food production is the primary issue to be handled, which also demands large amounts of water. However, water quality and quantity directly determine food production. As such, whether we can solve water-related problems will affect the development and sustainability of China.

## 3. An Adaptive Governance Frame for Water Management

### 3.1. The Weakness of the Current Frame

The current water resource management frame did solve some water-related problems. Some existing problems are becoming more and more serious. What’s more, more problems are looming on the horizon and the current frame has not been ready for climate change and human activities.

Due to China’s imbalanced development, more region-specific policies have not been made by considering the local realities. For instance, in water-shortage areas, more water-saving policies, such as drop irrigation, rainwater collection, and reuse, and treated wastewater reuse, should be encouraged, while in water-rich areas, more water quality improvement policies, such as advanced water treatment technologies and more stringent water quality monitoring, should be adopted. Moreover, it is critical to enforce the regulations more effectively and efficiently. The current top- down approach has been filled with difficulties in dealing with the challenges that have appeared, let alone considering underlying dangers. The water management frame has been evolving for decades, but decision-making was only restricted in the government management department and decision-making institution, isolating the broad masses of common people. Meanwhile, water prices were set at an unreasonably low level, and in most cases, people did not attach importance to water saving. This management frame leaves a great negative effect; that is, much is unknown. People do not know about the water status, water allocation standard, water price, etc. This non-participation finally gives rise to the weak water-saving consciousness and low water-use efficiency.

For a long period, the central government has managed water services directly and provided water services to consumers at a low charge, or even free of charge in some underdeveloped areas [48]. This low price or free of charge led to a pervasive misunderstanding about water, the public thought they were at liberty to draw and use water, with the conception that water was free natural resource; anyone could use it at their will.

**Figure 6:**
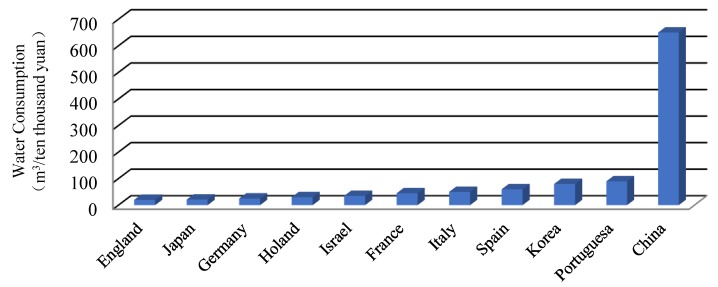
Water consumption per unit of GDP in some countries.

As shown in Figure 6, the water use efficiency is not high in China. The waste of water is serious, and the output GDP per water is only 1/518 compared to the world’s average, which is much different from that of developed countries.

Agricultural water consumption has been increasing over the past decade, accounting for nearly 65% of the total water consumption. What’s more, agricultural water-use efficiency is rather low. For instance, irrigation water efficiency is only 0.5, much lower than 0.8 in developed countries. Food production capacity is also very low, in China, only 1 kg of food can be produced per m^3^ of water, however, in the developed countries this figure can reach 2 kg, such as 2.35 kg in Israel [49]. The same situation can also be seen in industrial production. The repeat utilization rate of industrial water is about 50%, lower than the average level.

**Figure 7:**
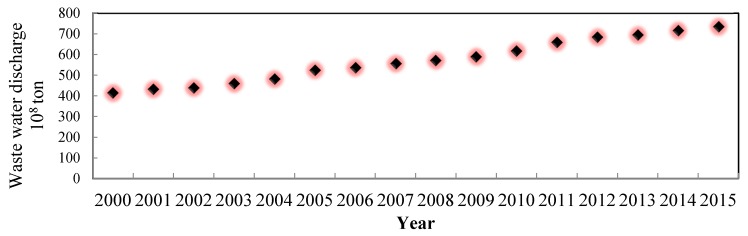
Wastewater discharge in China. (China Annual environmental statistics 2000–2015).

Water pollution has also become a crucial problem in China, and many rivers and lakes have been plagued by different levels of pollution. Wastewater discharge has grown to 735.3 × 10^8^ t in 2015, almost twice as much as 415 × 10^8^ t in 2000 (Figure 7). The growing discharge contaminated many lakes and rivers. In China, water quality is classified into five categories which can be described as ‘‘good’’ (Grades I, II, and III) and ‘‘poor’’ (Grades IV and V or V+), which cannot support drinking and swimming). V+ means the water has no economic or practical values any longer. As shown in Table 1, the ‘poor’ quality water (Grades IV and V or V+) in the Hai River accounts for 62.7 % of the length of the river, while in the Liao River and the Huai River, it is 54.7% and 46.7%, respectively. This number is alarming.

The Ministry of Water Resources takes charge of allocating and planning water holistically, and the Bureau of Environmental Protection is responsible for the protection of the water environment. From the perspective of results, this frame did not work out very well. When planning water utilization, if the principal of water protection is not included, the decision maker would look down on the water protection and try to maximize the economic benefits when it is allowed. As such, when the protection policies are enacted, no organization or individual is willing to follow. Once the water has been contaminated, it would take much more human and material resources to revert it.

As shown in Figure 4, watershed water resource protection bureaus are in the charge of both the state Bureaus of Environmental Protection and the Ministry of Water Resources. This could be unified, but watershed water resource protection bureaus are just affiliated departments of watershed water resource commission, restricting their entitlement. They can only carry out the work within the decision made by superior departments rather than take protection as a basic principle. This obstacle brings about the concession of water resource protection bureaus when making decisions.

There is a great overlap area between the watershed water resource commission and the provincial water resource department. Every provincial water resource department is located within a certain watershed. This overlap led to a bad consequence. On one hand, the seven watershed water resource commissions have a certain level of administrative power, but due to the setup of the provincial water resource department, their powers are greatly cut down. Although watershed water resource commissions could allocate and manage watershed water resources holistically, they still have difficulties in involving provincial water resource management, utilities, and protection. On the other hand, the provincial water resource department belongs to the management of provinces, which means they usually treat water as a tool to achieve official career, because in China, official achievements account for a large proportion when it comes to promotion. In many cases, the provincial water resource departments selectively ignore some plans and decisions made by watershed water resource commission to optimize the provincial benefits. For instance, the upstream province would overlook the integrated water consuming plan made by the watershed water resource commission and over-pump water from rivers to develop local economies. This activity sometimes leads to a water shortage in some downstream provinces; sometimes only crop yield was affected. When it gets worse, domestic water could become a problem [50].

Inadequate public participation, poor management of water resources by management agencies, shortage protection for emergencies, and little communication among river basin management stakeholders are all prominent problems in water resource management. The existing system lacks adaptability, which makes the decision unreasonable, and not comprehensive. It is only a top-down policy implementation mechanism, which lacks the adjustment to the feedback information to increase the adaptability. However, adaptive water resource management can solve these problems, which also needs the supports from appropriate institutions.

### 3.2. An Adaptive Governance Frame for China

Sustainable management of complex adaptive systems is challenged by different temporal, spatial, and social scales, nested hierarchies, inevitable uncertainty, multidimensional interactions, and emergent properties [51,52,53]. According to the weakness of the current frame, the current so-called command-control approach has been increasingly criticized as it insulates itself from the needs and circumstances at the lower levels in response to changing environmental conditions [38,39]. It generates what one may refer to as lock-in situations, a term introduced in economics to describe the dominance of established technologies despite inferior performance due to path-dependence. This kind of planning frame is vulnerable to surprise and crisis because management institutions become rigid.

The increasing awareness of the impacts of climate change has led to the insight that water management must become more flexible to deal with uncertainties and surprises [6]. The eco-hydrological system in China is undergoing dramatic changes in recent decades, due to climate change and the construction of cascade dams for power production [54]. In the last decades, a growing scholarship has promoted the development of adaptive governance frames to enhance institutional capacity and rationalize water management policies and practices [42].

The adaptive governance frame is generated from the complex adaptive system (CAS), a complex, nonlinear, self-organizing system that can adapt to a changing environment [44]. The most prominent feature of the adaptive governance frame is the ability to learn. Such a system can learn from the experience and avoid making the same mistakes. A CAS consists of many essential elements (i.e., agents) interacting with each other constantly according to specific rules. These agents keep interacting, learning, and gaining experience from such behavior. For example, the immune system, the stock market, the economy, and so on. Modular system structure and decentralized control lead to more adaptiveness and robustness of a system [55].

Adaptive governance is used to deal with resource management problems that are not easy to understand and difficult to control. Compared with traditional management methods, adaptive governance is mostly characterized by high uncertainty and low controllability. The fundamental differences between adaptive governance methods and traditional management lie in that adaptive governance changes from trial and error, and managers constantly adjust strategies to meet the needs of management with the uncertain external environment. The management process is ring-shaped because of the feedback. The traditional management mode generally adopts the administrative instruction and does not test the system complexity, which causes the lag phenomenon in management, so the management process is linear.

**Figure 8:**
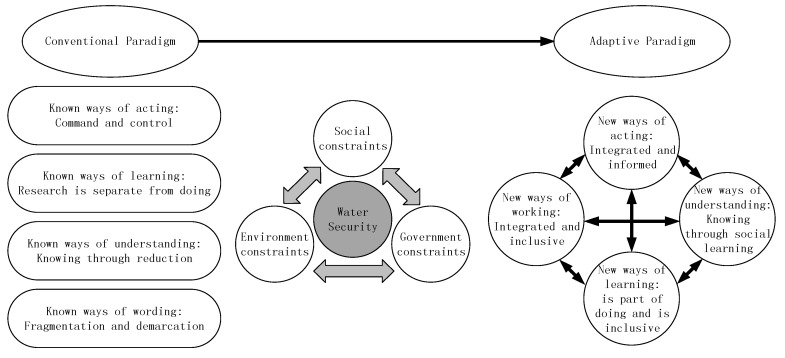
Comparison between conventional paradigm and adaptive paradigm.

In China, within an adaptive paradigm, the concept of water security would be continuously reviewed and reconstructed to meet the requirements of a continuously changing world, rather than being considered as a predetermined goal or endpoint (see Figure 8) [56].

The current command-control frame of water resource management in China is a static approach, which has helped to reduce life and property losses and achieve stability in socio-economic development. However, the development and utilization of water resources are still a problem in China. The country may face several future challenges related to water resources management: (1) Human activities and population growth: As the population grows, it will increase the water consumption and water environment. Human activities will increase domestic water demand and water pollution [17,57]. (2) Climate change: A number of studies also suggested that climate change will improve the water resource management requirements in China [58,59]. (3) Water scarcity: Continuing destruction of freshwater resources and growing water demand have increased the unevenness in water supply and demand in China in recent years [60]. Many of the rivers in northwest China are already under stress due to a high degree of exploitation. However, the challenges will exist in the water resources management of China to accomplish sustainable development [54]. Hence, an adaptive water resource management frame will be an important subject in China.

#### 3.2.1. Learning Cycles in China an Adaptive Governance Frame for China

Adaptive governance can be described as a systematic process, continually improving policies and activities by learning from the outcomes of actions and activities. Akamani et al. [37] proposed adaptive governance as a unifying framework for informing policies aimed at promoting the conservation of transboundary water resources in an increasingly unpredictable future, as well as preparing the coupled social-ecological system to respond to unpredictable drivers of change. Akamani [61] highlights four attributes of adaptive water governance: (1) reintegrating humans into nature; (2) integrating diverse sources and types of knowledge; (3) promoting adaptive and integrative management goals; and (4) using polycentric institutions and analytic deliberation processes. Pahl-Wostl and Lebel et al. [28] defined the governance system as a broader notion, which encompasses structural features and transient processes at both rule making and operational levels, which could help formulate and implement water policy.

**Figure 9:**
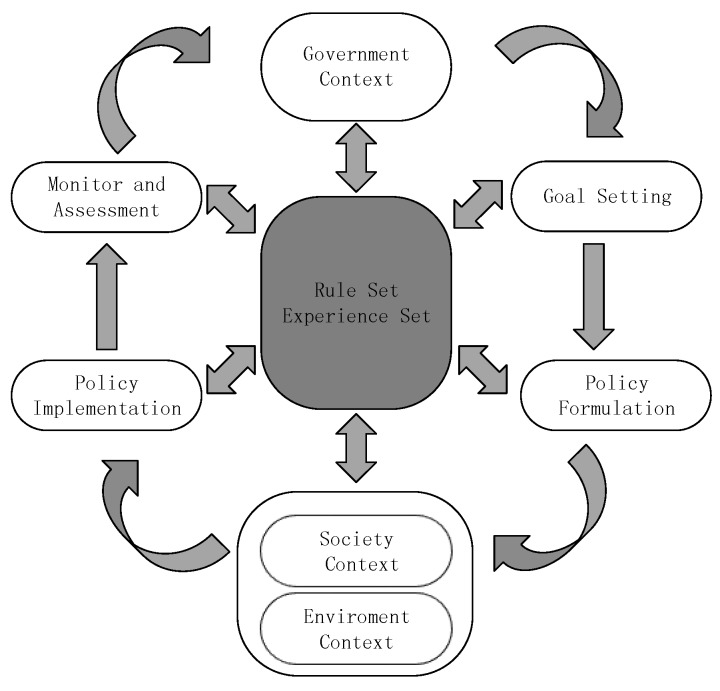
Learning cycle related to policy cycle. Government context module, society context module, environment context module refers to the participation of relevant agents.

According to the literature and our practical investigation, the paper thinks the kernel of adaptive governance is the learning cycle, which can be summarized in Figure 9. The whole process contains six steps in water resources management in China: government context, goal setting, policy formulation, social context and environmental context, policy implementation, monitoring, and assessment. The learning cycle is embedded in two aspects:

(a) The continuous loop:

The whole process is a continuous loop. All the policies are not fixed, which allows change and adjustment. The current frame is based on the optimal mode, optimizing the predetermined goal according to known knowledge and information. Once determined, change is less likely to happen. Data integrity is a controversial topic; many researchers queried the possibility of data integrity, claiming that the water resource system is highly sensitive and nonlinear. Even if it is possible to collect all the data, this massive workload is unable to finish under current conditions. According to CAS, the water resource system is of high complexity and unpredictability. Based on the aforementioned two reasons, optimization is just an ideal model, which can never be achieved. Implementation will gradually deviate from the original plan, and once serious emergencies take place, the original plan will lose efficacy.

The continuous loop could avoid this problem properly. However, it does not mean it would not make any mistakes; due to the continuous process rather than a one-way process, the identified problem can be fixed in time. Monitoring and assessment are other aspects. Including a broader set of stakeholders gives access to different kinds of knowledge which is vital for a full assessment of a resource governance problem and for finding innovative solutions for dealing with it.

In terms of water resource management in China, this loop represents the management policies that will be continuously monitored and evaluated with the influence of various participants, environment, and society during the implementation process of the relevant policies of water resources management, thus forming a more optimized management decision.

(b) Rule set and experience set:

In the adjustment, the rule set and experience set play a shaping role. It is an aggregation of gained experience and tested rules. The continuous loop enables the trial and error while the rule set and experience set are established to avert committing the same error twice.

Every part or corresponding agent could add their new insights, novel ways into the set, and extract relevant knowledge to prop up their decision. It is an evolutionary and complementary process. For example: without the rule set and experience set, the continuous loop may make the same mistakes again and again; without the continuous loop, the rule set, and experience set cannot be expanded and completed.

In China, there are many water management practices that can be considered as a rule or experience. Managers should classify these management rules and experiences into the form of a smart library. In the process of water resources management, according to these practices, policy and water resources management can be better implemented. These rules and experiences are both the basis for progress in water management and the standard for testing water management levels, which are constantly evolving to form new rules and experiences.

#### 3.2.2. Participation

Increased interaction between the public and society with policymakers is conducive to the improvement of the decision-making process. Interactions of the participation can change the proposed, discussed, and implemented policy recommendations in detail. As shown in Figure 8, The society and environment context are part of the whole loop. It is a reflection of participation. In the current management frame, the entire approach is considered to focus predominantly on biophysical data, neglecting dynamic aspects such as adaptive capacity, vulnerability, and resilience [14,15]. In most circumstances, decision-makers will list several critical parameters on behalf of society and the environment.

This isolation is the culprit of many problems discussed in Section 3, such as water wasting, flooding, and water quality degradation. There are results from an evaluation of the project that showed that participants had become better informed about climate change issues (e.g., flooding and water quality degradation), and also had an enhanced ability to analyze climate change issues from multiple perspectives [61].

The lack of social context (i.e., the involvement of citizens and social organization) augments the possibility of uncertainty; Newig J. et al. [62] claimed that the participation of interested parties could reduce uncertainties in the implementation phase by reducing the likelihood of unexpected resistance. Nowadays, the segregation of the public in policy making sets up a roadblock in the implementation in China. The Chinese public view an issue from their limited perspective and are unwilling to follow the rules due to the deficiency of requisite information. In China, water resources are seriously wasted, so raising public awareness of water resources protection and the public participation in water resources management is necessary. Water conservation, water protection, and water cherishing from everyone are also beneficial to water resources managers. The public can solve various water resources problems spontaneously and timely.

### 3.3. Specific Frame of Adaptive Water Resources Management in China

The paper proposes the adaptive governance as a conceptual foundation for adaptive and integrated water resource management, thereby providing a framework for integrating the stakeholder participation into the governance of water resources while simultaneously dealing with uncertainties [63].

**Figure 10:**
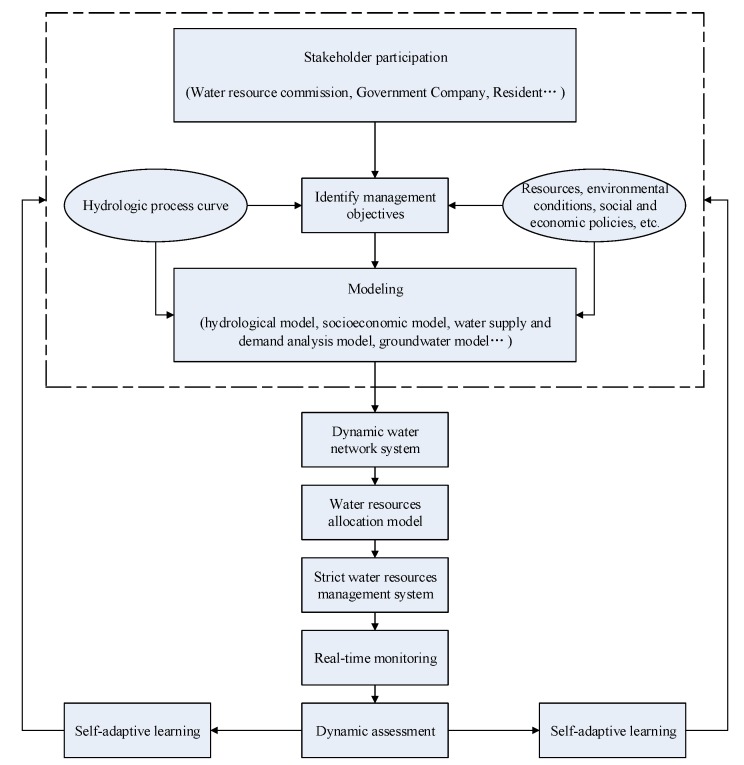
Water resources adaptive governance frame in China.

According to the framework of adaptive governance of water resources, a frame of adaptive governance of water resources is established, as shown in Figure 10. This management frame is composed of several stakeholders who jointly set targets for water resource management based on complex factors, such as hydrological conditions, resources, environment, and socio-economic conditions in the region, and make use of hydrological, socio-economic, and optimal allocation models of water resources, etc., to verify and improve this goal. After making the phased management objectives, the frame takes water system construction, a strict water resource management system, a river-belonging system, and other measures to guide and strengthen regional water resource utilization and protection. Because of the effect of the implementation of various measures, the frame adopts unique and integrated real-time monitoring and dynamic assessment to contrast assessment results with management objectives and carry out self-adaptive learning to determine adjustment measures and plans, then take adjustment measures and plans as input conditions for the next stage of decision-making. Finally, relevant groups carry out a new round of water resource management objectives, considering other factors.

According to the adaptive governance framework, no matter whether it be the manager or the water using sectors, even each water user, they should participate in the process of water resources adaptive governance to improve the awareness of water conservation. This type of governance framework is appropriate for facilitating the current emphasis on multiparty stakeholders in water resource governance and for preparing the self-adaptive learning for the dynamic change in response to the uncertainty in the water network system.

In January 2017, President Xi Jinping issued "Each river is going to have a river chief" in the New Year message, and in June 2018, the river chief system has been established in all 31 provincial regions on the Chinese mainland, with 300,000 officials appointed chiefs to protect water bodies. The river chief system is a significant decision considering the adaptive governance framework. River chiefs are responsible for organizing and leading the management and protection of relevant rivers and lakes at various levels and assessing the completion situation of objectives and tasks. The river chief governance in water resources plays an important role in promoting the harmonious coexistence between human beings and nature and accelerating the construction of ecological civilization. It is a major adaptive governance innovation that solves some water problems in China and guarantees water security.

According to current regulations, the river and lake management system in China is implemented both at the river basin level and administrative region level, and is carried out both in a centralized manner and a hierarchical manner. The water administrative department in the State Council is responsible for the centralized management and supervision of national water resources. It is the leading authority for riverways (including lakes) and it sets up river basin commissions for important rivers and lakes, as determined by the State Council. Local administrative departments in charge of water resources above the county level are responsible for the centralized management and supervision of water resources within the administrative region according to the stipulated duties, and are the authorities for riverways (including lakes) in the respective administrative regions. Relevant government departments at various levels are responsible for relevant work on water pollution control, water environment treatment, and water ecological protection according to their assigned responsibilities.

After comprehensively implementing the adaptive governance of the River Chief System, the shorelines of rivers and lakes have gradually recovered, the environmental pollution of some rivers and lakes have basically been eliminated, and the water quality of some rivers and lakes has significantly improved, and the scenes of smooth rivers, clear water, and green shores have begun to appear.

## 4. Discussion

There is a complex relationship between governability, governance, and public management. All three are participants in water resources management, and efficient water resource management is inseparable from their coordination and cooperation. Governability provides legal and policy guarantees for water resources governance, but public management is also critical to water resources governance; the main body of governance must be the public institutions of society, and the government manages water resources through public institutions. The main body of governance can be either public or private institutions, or cooperation between public institutions and private institutions. As such, governance is the cooperation between governability and civil society, the cooperation between government and non-government, the cooperation between public agencies and private agencies, and the coercive and voluntary cooperation. Governance is a broader concept than the government, which has more participants. If the government wants to manage the water resources efficiently and in an orderly way, it cannot be separated from a government rule and provided with legal and regulatory support, and it cannot be separated from public management and protection. As such, the paper makes relevant recommendations from the following aspects.

### 4.1. Water Resources Managers

Water resource managers should be fully familiar with the previous management cases and experiences, considering social, economic, environmental, and other factors comprehensively. The technicians grasp the real-time monitoring data of water resources and establish intelligent water resource management platforms and systems to help managers make reasonable goals and adjust them in real time based on other impacts during water resource management.

### 4.2. Water Using Sectors

When using water resources, all departments should raise awareness of the rational use of water resources, improving water use efficiency, and combine their water use condition with other departments, recording water usage in real time and providing supervisory feedback to achieve the sharing of water resource information at different levels. According to the external adjustment of the department’s water use, the water management framework can achieve a reasonable allocation of water resources and maximize the overall benefits.

### 4.3. Water User

As the smallest unit of water resources use, each citizen should raise awareness of water conservation, save water, and call on people around to save water resources, protect the water environment, and implement policies related to water resources management, such as the river belong system, etc. If there are some water resource problems, the users should find them and deal with them in time.

## 5. Conclusions

With more and more demand for water in agriculture, industry, citizen consumption, and ecology, the current water cannot support it anymore. Coupled with the water environmental pollution, the situation gets even worse. In the literature, we analyzed the water resource management status in China and emphasized the necessity to take complexity into account in the resource management frame and develop adaptive methods.

In this paper, the weakness of the current management frame was analyzed. Though the current command-control management has been amended and corrected many times, the disadvantages have become apparent. It gave rise to the isolation of the public, the institutional inertia, the separation of water utilization and protection, etc. The water resource system is a complex adaptive system (CAS), which is a complex, nonlinear, self-organizing system, hence, we introduce adaptive governance to surmount traditional deficiencies.

We constructed a feasible and alternative frame for water resource management, which takes learning and participation into consideration. The current command-control management should be replaced by adaptive governance. Adaptive governance is based on the learning cycle, which could learn from experience and adjust according to the actual situation. A fixed and invariable way is unable to deal with emergencies and surprises. The participation of different agents, such as managers, water using sectors, and water users provide more comprehensive knowledge in decision making from water managers to water sectors to each water user.

The advantages of adaptive water resource management are 1) the cooperation of different stakeholders, 2) an effective organizational framework, 3) the strengthening of the decision-making process. In short, the adaptive governance frame is aimed at the complex water resources system, carrying out a repeated cycle of identifying, monitoring, assessing, coping, and adjusting a series of actions. The adaptation of the water resources system is accustomed by adjusting the water resource management mode and allocation plan continuously, and then it can promote the development, utilization, and protection of water resources, and constantly adapt to the coordinated and sustainable development from economic, social, ecological, and other aspects.

## Figures and Tables

**Table 1:** Water quality of major ten rivers of China in 2016 (China environmental state bulletin).

Name	Grade I–III (%)	Grade IV, V (%)	Grade V+ (%)
The Yangtze River	82.3	14.2	3.5
The Yellow River	59.1	27	13.9
The Pearl River	89.6	6.7	3.7
The Songhua River	60.2	33.3	6.5
The Huai River	53.3	39.5	7.2
The Hai River	37.3	21.7	41
The Liao River	45.3	39.6	15.1
The Zheminpian River	94.4	5.6	0
The River of northwest	93.5	6.5	0
The River of southwest	90.5	9.5	0
